# Photobiomodulation Acutely Augments Resting Metabolism in Women with Obesity

**DOI:** 10.3390/nu17213357

**Published:** 2025-10-25

**Authors:** Massimo De Nardi, Silvia Allemano, Marta Buratti, Eva Conti, Luca Filipas, Daniel Gotti, Livio Luzi, Roberto Codella

**Affiliations:** 1 Department of Neuroscience, Rehabilitation, Ophthalmology, Genetics, Maternal and Child Health, University of Genoa, 16132 Genoa, Italy; denardiwbc@gmail.com; 2 Department of Biomedical Sciences for Health, Università Degli Studi di Milano, 20133 Milan, Italy; silviaallemano@outlook.com (S.A.); luca.filipas@unimi.it (L.F.); daniel.gotti@unimi.it (D.G.); livio.luzi@unimi.it (L.L.); 3 Department of Endocrinology, Nutrition and Metabolic Diseases, IRCCS MultiMedica, 20138 Milano, Italy

**Keywords:** red-light therapy, energy metabolism, obesity treatments, weight management, mitochondrial efficiency

## Abstract

**Background/Objectives**: Photobiomodulation (PBM) is a non-invasive, low-level laser treatment shown to improve insulin resistance, glucose metabolism, and obesity-related inflammation. This study examined whether PBM could acutely enhance mitochondrial efficiency and energy metabolism in women with obesity. **Methods**: In a randomized, crossover within-subject design, 16 women with obesity (43 ± 5 years; BMI: 36 ± 4 kg/m^2^) and 16 sedentary normal-weight women (43 ± 5 years; BMI: 22.7 ± 2 kg/m^2^) underwent PBM (front and back exposure; red light, 633–660 nm; NIR, 850–940 nm) and a sham stimulation (SHAM), as a control, for 12 min. Resting energy expenditure (REE) was assessed via indirect calorimetry before and after exposure. Secondary measures included skin autofluorescence, heart rate, blood pressure, profile of mood states, rate of perceived exertion (RPE), and flexibility. Diet and physical activity were controlled. **Results**: A 2 × 2 × 2 ANOVA revealed a significant group × time interaction (F_3,60_ = 3.054, *p* = 0.03) and a main effect of time (F_1,60_ = 10.88, *p* = 0.001). Women with obesity showed a significant increase in REE post-PBM compared to pre-PBM (+9.3%, 1624 ± 314 vs. 1486 ± 327 kcal/day; *p* < 0.001), with no change in the respiratory exchange ratio. Additionally, RPE decreased and flexibility improved in both groups following PBM. Front and back skin temperatures increased significantly post-PBM, with greater changes observed in the back versus the front. **Conclusions**: These preliminary findings indicate that PBM acutely enhances energy utilization efficiency in women with obesity, increasing resting energy expenditure without modifying substrate oxidation. PBM may represent a promising non-invasive adjunctive strategy for improving the metabolic health of obese individuals.

## 1. Introduction

Obesity is a multifactorial condition characterized by excessive adiposity, low-grade systemic inflammation, and metabolic dysregulation, including insulin resistance and impaired glucose homeostasis [1,2]. The global prevalence of obesity continues to rise, disproportionately affecting women, who often experience additional cardiometabolic impairments and reduced quality of life [2]. Among the major contributors to obesity are dietary excess, poor nutritional quality, and sedentary behavior, which together promote a chronic imbalance between energy intake and expenditure [3]. Traditional therapeutic approaches, including lifestyle modifications and pharmacological treatments, often show limited long-term success, underscoring the need for innovative, non-invasive adjunctive therapies that target metabolic dysfunction.

Photobiomodulation (PBM) is a non-invasive technique that uses red to near-infrared (NIR) light (600–1100 nm) to modulate biological processes without generating heat or tissue damage [4]. At the cellular level, PBM is thought to act primarily on mitochondrial chromophores such as cytochrome c oxidase within the electron transport chain [5]. Absorption of photons at these wavelengths enhances electron transfer, increases mitochondrial membrane potential, and can promote adenosine triphosphate (ATP) synthesis [6,7]. These bioenergetic changes trigger downstream signaling pathways that may improve cellular function, redox balance, and tissue metabolism [8,9]. Emerging evidence suggests that PBM may favorably influence several physiological systems relevant to obesity and metabolic health, including muscle performance [10], glucose metabolism [11,12], inflammation [13,14], and vascular function [15]. Notably, its effects appear more pronounced in conditions of metabolic stress [16,17], such as aging and obesity, where mitochondrial and endothelial efficiency are often compromised. Experimental and clinical findings indicate that PBM exposure can enhance tissue oxygen utilization [18,19,20], modulate insulin sensitivity [11], and increase overall metabolic activity.

Despite these promising insights, the acute effects of PBM on resting energy metabolism in individuals with obesity remain poorly understood. Resting energy expenditure (REE) constitutes the largest component of daily energy balance and reflects basal metabolic processes closely linked to mitochondrial and cellular efficiency. Understanding whether PBM can acutely modulate REE—independently of changes in substrate oxidation—may provide valuable information about its potential as a metabolic support tool.

Therefore, the present study aimed to investigate the acute effects of PBM on resting energy metabolism in women with obesity using a randomized, controlled design. Specifically, we examined whether a single 12-min exposure to combined red and NIR light would acutely increase REE in comparison with a sham control while also assessing secondary physiological and perceptual responses.

## 2. Materials and Methods

### 2.1. Participants

Normal-weight and middle-aged women with obesity were recruited for this study. The sample size estimation was based on effect sizes reported in our previous studies using similar protocols and outcome measures [21,22]. Specifically, by using a sample-sized determination software package (G * Power 3.1.9.2, Düsseldorf, Germany), with a statistical power of 0.8, a probability level of 0.05, an effect size *f* of 0.26, a total of 32 women were volunteered to participate in this study. The study design and procedures were approved by the local research ethics committee of the Università degli Studi di Milano (n° 52/20) and followed the ethical principles for medical research involving human participants set by the World Medical Association Declaration of Helsinki. Each subject was informed of the procedures and risks before giving a written in-formed consent to participate in the study.

### 2.2. Experimental Design

**Figure 1:**
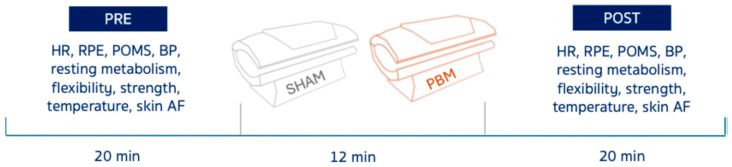
Scheme of the experimental protocol. Heart rate (HR), rate of perceived exertion (RPE), profiles of mood states (POMS), blood pressure (BP), and autofluorescence (AF).

This study used a randomized, crossover design. Each participant completed two experimental sessions–PBM and SHAM–in randomized order, separated by at least one week to minimize carry-over effects. Sixteen women with obesity and 16 normal-weight women (Table 1) underwent both PBM (front & back exposure) and control (SHAM) for 12 min (Figure 1). Resting energy expenditure (REE), temperature (front & back), skin autofluorescence (AF), heart rate (HR), blood pressure (BP), profile of mood states (POMS), forearm flexors strength, hamstrings and lower back linear flexibility, and rate of perceived exertion (RPE) were assessed pre- and post-exposure (Figure 1).

On visit 1, before any intervention, a physician conducted a physical examination of the subjects to exclude any contraindication to PBM, such as pregnancy, epilepsy, active skin cancer and photosensitivity [23]. Pharmacological therapy was assessed before the study. People undergoing any pharmacological therapy on a regular basis or taking medication influencing metabolic homeostasis were excluded. Also, people who had been exposed to PBM procedures within the last 6 months, smokers, alcohol abusers, following specific diets or taking supplements that may affect the metabolic rate, were also excluded from the trial. Subjects’ body weight and height were measured to the nearest of 0.1 kg and 1 mm respectively, by using a segmental body composition monitor scale (Tanita BC 601-F, Tanita corporation, Tokio, Japan), and stadiometer device. BMI was calculated for each individual as weight (kg)/height (m)^2^ (Table 1).

Throughout the entire duration of the study, participants were encouraged to maintain their physical and dietary habits. As to the energy balance not deriving from REE, subjects were screened for physical activity levels (IPAQ questionnaire) [24] and caloric and dietary intake (EPIC questionnaire) [25,26].

*Red-light therapy bed.* The device (LED PRO, CTN, Helsinki, Finland) is shaped like a bed (dimensions: 192 × 85 × 95 cm) and equipped with 28,512 LEDs emitting red light (λ = 633–660 nm) and near-infrared light (NIR, 850–940 nm) in continuous mode, without interruptions. This configuration produces an irradiance of 129 mW/cm^2^ and a fluence of 92.9 J/cm^2^ at a distance of 5 cm from the supine contact surface (Supplementary Figure S2B), and 5–10 cm from the anterior body surface (Supplementary Figure S2A), depending on thoracic–waist circumference. The device is equipped with a cooling system comprising three fans. Participants underwent either PBM or SHAM during visit 1 and visit 2, scheduled one week apart. Each visit took place in the morning between 09:00 and 10:30. During the sessions, participants lay supine on the treatment bed, wearing only underwear and protective goggles, as recommended by the manufacturer. Each session lasted 12 min. All participants tolerated PBM well, and no adverse events were observed during or after the sessions.

To minimize potential placebo effects, the SHAM condition was performed in the same setting as PBM, with the lights switched off while the cooling fans and acoustic countdown signal remained active. Participants continued to wear protective goggles throughout the procedure.

Before and after any condition, the following measures were taken:

*Resting energy expenditure measurements.* REE was assessed using a validated indirect calorimeter (Q-NRG; Cosmed, Rome, Italy) before and after PBM application. Participants were instructed to follow standardized lifestyle and dietary procedures 24 h prior to the measurement, including fasting, avoiding physical activity, and refraining from coffee consumption. They also received specific guidance to ensure proper execution of the procedure. During testing, participants remained clothed and lay supine on a medical examination bed for 10 min before an isolating plexiglass canopy was placed over their head. A further 10-min acclimatization period allowed for adaptation to the device and stabilization of experimental parameters. Subsequently, a 20-min recording was conducted, during which participants remained motionless, refrained from closing their eyes, and kept their legs uncrossed. Respiratory gas exchanges ($\dot{V}$CO_2_ and $\dot{V}$O_2_) were measured continuously. REE was calculated using the device’s dedicated software (OMNIA, Cosmed, Rome, Italy), and the Respiratory Exchange Ratio (RER) was determined as the ratio of $\dot{V}$CO_2_/$\dot{V}$O_2_.

*Lumbar–hamstring extensibility.* Flexibility was assessed using the standardized sit-and-reach test, performed with a Flex-Tester box (Cranlea, Birmingham, UK), in accordance with ACSM guidelines. Participants were barefoot, seated with hips and knees fully extended, and feet placed flat against the box. They were instructed to reach forward along the measuring scale as far as possible and to hold the maximal position for at least 2 s [27]. The task was repeated two times. The better of the two trials from each time point was taken for further statistical analysis.

*Strength.* Each participant familiarized themselves with a portable JAMAR Hydraulic Hand Dynamometer (Sammons Preston Rolyan, Nottinghamshire, UK) using the dominant hand. The device was adjusted individually to fit the hand and permit flexion at the metacarpophalangeal joints. Handgrip strength was expressed in kilograms. Participants were seated upright with their back supported and feet flat on the floor [28]. The testing position was standardized: shoulder adducted and neutrally rotated, elbow flexed at 90°, forearm and wrist in neutral, and the hand aligned with the forearm while holding the dynamometer vertically on its base [29]. After familiarization with three submaximal 5-s contractions, participants performed the test, which demonstrated excellent reliability (α = 0.946).

*Skin Autofluorescence*. To assess skin advanced glycation end products (AGEs) accumulation, non-invasive measurement of skin autofluorescence (AF) on elbow flexors were performed with an excitation light source of 300–420 nm. An AF reader (AGE Reader mu, DiagnOptics, Groningen, The Netherlands) illuminates approximately 1 cm^2^ of skin on elbow flexors with an excitation light source of 300 to 420 nm [30]. AF is calculated by dividing the average light intensity emitted per nm over the 420- to 600-nm range by the average light intensity emitted per nm over the 300- to 420-nm range. All measurements were performed at room temperature in a dark environment.

*Thermography.* Whole-body thermal images were acquired within 1 min before and after each light therapy exposure using a thermal imaging camera (E54, Flir Systems, Danderyd, Sweden), following established standards for infrared medical imaging [31,32,33]. The device had a resolution of 320 × 240 IR pixels, a spectral range of 7.5–14 μm, and a measurement range of −20 °C to 650 °C (accuracy ±2 °C). As previously reported [34,35], measurements were conducted in a dark room with only the subject and evaluator present. The camera was switched on and calibrated at least 10 min before use, and procedures adhered to the Thermographic Imaging in Sports and Exercise Medicine (TISEM) checklist [36]. Eight regions of interest (ROIs) were analyzed (right/left thigh, leg, arm, and upper/lower trunk). Each ROI was assessed bilaterally (anterior and posterior), yielding 16 ROIs per participant. An experienced evaluator (>5 years) defined ROI boundaries, and mean temperatures were extracted using dedicated software (Thermacam Researcher Pro 2.10, Flir Systems, 2015) with emissivity set to 0.98. Participants were instructed to remain still and avoid behaviors that could alter skin temperature.

*Physiological and perceptual measures.* Heart rate was recorded using a chest strap heart rate monitor (H9, Polar, Kempele, Finland), measured before and after, as well as every 3 min during the light therapy exposure. Rating of perceived exertion was assessed using Borg’s CR10 scale [37]. BP was measured in a seated position using a digital sphygmomanometer. Mood was evaluated using the Brunel Mood Scale (BRUMS) developed by Terry et al. [38], based on the Profile of Mood States [39] and adapted for the Italian population [40]. The questionnaire consists of 24 items divided into six mood-related subscales (Depression, Fatigue, Vigor, Confusion, Anger, Tension). Participants rated each item on a 5-point Likert scale (from 0 = not at all, to 4 = extremely) in response to the prompt: “How do you feel right now?”.

### 2.3. Statistical Analysis

All data are presented as mean standard ± deviation (SD). The assumptions of normality and sphericity were checked using the Shapiro–Wilk test and the Mauchly test, respectively. The test–retest reliability of the two pre-stimulation measurements (pre-SHAM; pre-PBM) was measured using an intraclass correlation coefficient (ICC, Cronbach-α) and interpreted as follows: α ≥ 0.9 = excellent; 0.9 > α ≥ 0.8 = good; 0.8. > α ≥ 0.7 = acceptable; 0.7 > α ≥ 0.6 = questionable; 0.6 > α ≥ 0.5 = poor [41]. Subjects’ characteristics were compared with a two-tailed unpaired *t*-test (Table 1). A 2 × 2 × 2 repeated-measures analysis of variance (RM-ANOVA) with factors of time (pre, post); group (normal-weight, obesity, between-subject); condition (SHAM, PBM, within-subject) was performed for any other variable. Šídák’s or Tukey’s post-hoc tests were used for pairwise comparisons following significant main or interaction effects. Individual-level effect sizes (Cohen’s *d*) were calculated to quantify each participant’s response to the intervention. Effect sizes were interpreted as follows: 0.0–0.2 = trivial, 0.2–0.6 = small, 0.6–1.2 = moderate, 1.2–2.0 = large, and >2.0 = very large [42]. To test the association between the change in resting energy expenditure (ΔREE) and the change in skin temperature (ΔT) across groups and conditions, we computed Pearson correlation coefficients (r) separately for each group × condition cell (obesity–PBM; obesity–SHAM; normal-weight–PBM; normal-weight–SHAM). For all analyses, a *p* value less than 0.05 was considered statistically significant. The data analysis was performed using the GraphPad Prism software (GraphPad Software, Version 11, San Diego, CA, USA), and Excel version 16.32 for Mac (Microsoft, Redmond, WA, USA).

## 3. Results

Subjects’ anthropometrical and energy characteristics at baseline are summarized in Table 1. Both groups were sedentary according to IPAQ questionnaire. No significant difference was found as to the estimated dietary intake between groups (Supplementary Table S2).

The reliability of the pre-stimulation measurements was excellent for both normal-weight participants (α = 0.91) and women with obesity (0.93).

**Figure 2:**
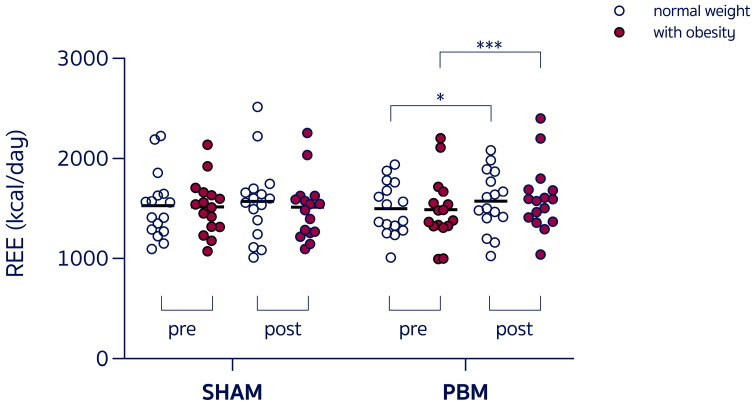
Resting energy expenditure (REE) during the experimental sessions under control condition (SHAM) or red-light therapy (PBM), in different-weight women. * *p* = 0.02; *** *p* < 0.001.

**Figure 3:**
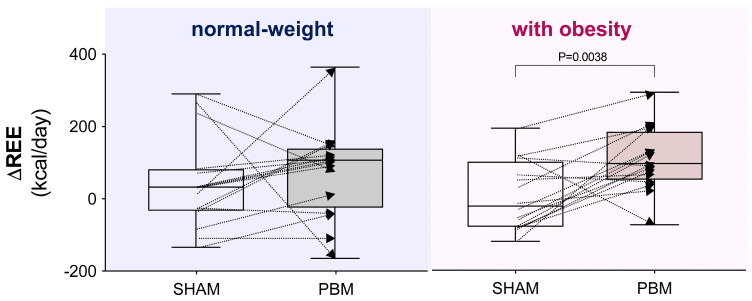
Interindividual variability of the delta (Δ post–pre) resting energy expenditure (REE) under different experimental conditions. *Box-and-whisker plot:* the box represents the interquartile range (25th–75th percentiles), and the whiskers extend to the minimum and maximum values, with the line inside the box indicating the median. In the right panel, the comparison is significant according to the Wilcoxon matched-pairs signed-rank test.

A 2 × 2 × 2 RM-ANOVA revealed (Table 2):

- A group × time interaction (F_3,60_ = 3.054, *p* = 0.03) and a main effect of time (F_1,60_ = 10.88, *p* = 0.001), with REE increased in women with obesity post-PBM compared to pre-PBM (+9.3%, 1624 ± 314 vs. 1486 ± 327 kcal/day, *p* < 0.001, Figure 2), with RER unchanged. Individual variability in the SHAM condition was trivial for both normal-weight participants (Cohen’s *d* = 0.117) and participants with obesity (Cohen’s *d* = 0.021), whereas the effect size in response to PBM was small in normal-weight participants (Cohen’s *d* = 0.265) and moderate in women with obesity (Cohen’s *d* = 0.640).
- A group x time interaction (F_3,60_ = 20.59, *p* < 0.001) and a main effect of time (F_1,60_ = 37.59, *p* < 0.001), with VO_2_ increased in women with obesity post-PBM compared to pre-PBM (+7%, *p* < 0.001).
- A main effect of time (F_1,60_ = 17.04, *p* = 0.0001), with HR decreased following the SHAM condition in both normal-weight women and women with obesity.
- A main group effect for systolic pressure (F_3,60_ = 13.19, *p* < 0.0001), with significantly increased values in women with obesity with respect to normal-weight counter peer (*p* < 0.001).
- A main effect of time (F_1,60_ = 21.1, *p* < 0.0001), with POMS scores improved in both groups after both conditions.
- A group x time interaction (F_3,60_ = 9.000, *p* < 0.0001), a main effect of group (F_3,60_ = 2.791, *p* = 0.04) with increased values in women with obesity compared to normal-weight women (*p* < 0.0001), and a main effect of time (F_1,60_ = 32.00, *p* < 0.0001) with RPE decreased in both normal-weight women and women with obesity, after PBM condition.
- A main group effect for skin AF (F_3,60_ = 10.48, *p* < 0.001) with significantly increased values in women with obesity with respect to normal-weight peers (*p* < 0.001).
- A group x time interaction (F_3,60_ = 8.835, *p* < 0.0001), a main effect of group (F_3,60_ = 4.095, *p* = 0.01) with better scores in lean subjects with respect to women with obesity (*p* < 0.05), and a main effect of time (F_1,60_ = 45.34, *p* < 0.001), with flexibility improved in both normal-weight women and women with obesity after the PBM condition.
- A group × time interaction (F_3,52_ = 75.17, *p* < 0.0001), a main effect of group (F_3,52_ = 29.18, *p* < 0.0001), with higher front temperature values in lean compared to women with obesity (*p* < 0.05), and a main effect of time (F_3,52_ = 288.9, *p* < 0.0001) with an increase in front temperature post-PBM in both groups (*p* < 0.0001).
- A group × time interaction (F_3,52_ = 160.1, *p* < 0.0001), a main effect of group (F_3,52_ = 70.21, *p* < 0.0001) with higher back temperature values in lean compared to women with obesity (*p* < 0.02), and a main effect of time (F_1,52_ = 542.5, *p* < 0.0001), with an increase in back temperature post-PBM in both groups (*p* < 0.0001).
- Furthermore, a greater delta (post-pre) was registered in both groups (*p* < 0.0001) in back- versus front temperature (4.637 vs. 3.262 °C for normal-weight; 5.106 vs. 2.737 °C for women with obesity). The magnitude of this delta was significantly higher in women with obesity with respect to normal-weight women (2.368 vs. 1.375 °C, *p* < 0.0001).
- Considering the interindividual variability in the delta (Δ post–pre) REE, PBM was found to have a significantly greater impact on women with obesity compared with the SHAM condition (*p* < 0.01, Figure 3).
- No significant correlations were found between changes in resting energy expenditure (ΔREE) and changes in skin temperature (ΔT) at the anterior and posterior exposure sites, across groups and conditions (Supplementary Table S1).

## 4. Discussion

The present study provides preliminary evidence that acute photobiomodulation can transiently augment resting energy metabolism in women with obesity, as reflected by a ~9% increase in resting energy expenditure following a single 12-min exposure to red and near-infrared light. Notably, this effect occurred without any change in the respiratory exchange ratio, suggesting that substrate oxidation remained stable. These findings should be interpreted as short-term physiological responses rather than definitive evidence of metabolic enhancement. The study was designed as an acute, controlled experiment to explore immediate effects under standardized conditions, serving as a necessary preliminary step toward future longitudinal trials.

### 4.1. Potential Phyiological Mechanisms

While PBM has been hypothesized to influence mitochondrial bioenergetics through the absorption of red and near-infrared light by cytochrome c oxidase, leading to enhanced electron transport and ATP synthesis [6,7], our study did not include direct assessments of mitochondrial function. Therefore, any mechanistic interpretation should be considered speculative. The observed increase in REE may reflect an acute physiological response involving multiple factors, including improved microcirculation, mild thermogenic activation, or transient modulation of cellular energy processes.

The increase in back and front skin temperatures following PBM suggests that thermogenic effects might contribute to the higher energy expenditure observed. However, our additional correlational analysis showed no significant association between changes in temperature and changes in REE, supporting the independence of the metabolic response from surface heating. The greater temperature change in the posterior body region likely reflects the conformal characteristics of the PBM device rather than a physiological asymmetry (see Supplementary Figures S1 and S2).

Furthermore, red and near-infrared light exposure has been associated with enhanced peripheral circulation and endothelial function [18,43], potentially improving oxygen and nutrient delivery. These vascular responses may transiently facilitate metabolic processes at rest, contributing to the modest rise in REE observed after PBM.

### 4.2. Broader Physiological Implications

Secondary findings provide additional context for interpreting the systemic response to PBM. Improvements in flexibility and reduced ratings of perceived exertion after PBM may indicate local tissue or neuromuscular benefits, possibly related to enhanced perfusion, reduced stiffness [44], or relaxation effects. The improvement in mood state (POMS) observed across both PBM and SHAM conditions suggests that expectancy or relaxation may also play a role [45], consistent with previous evidence on the psychological effects of light-based interventions.

Importantly, PBM did not produce adverse cardiovascular effects. Despite baseline differences in systolic pressure between normal-weight and obese participants, no acute hemodynamic alterations were observed following PBM, supporting its safety and tolerability in this population.

### 4.3. Clinical Significance and Translational Potential

Although preliminary, the observed 9% increase in REE in women with obesity may hold clinical interest. Since REE represents the largest component of total daily energy expenditure, even small acute increases–if reproducible and sustained–could contribute to improved energy balance when integrated with diet and physical activity interventions. The stable RER suggests that PBM did not shift substrate preference, which might indicate a balanced enhancement of oxidative metabolism rather than preferential lipid or carbohydrate utilization.

Furthermore, the safety profile observed, with no adverse cardiovascular responses and improvements in perceptual outcomes, underscores the potential utility of PBM as a complementary strategy to existing lifestyle interventions. Its non-invasive nature and ease of application could make it particularly suitable for individuals who face barriers to engaging in structured exercise programs.

### 4.4. Limititations and Future Directions

This study has several limitations. The sample size was small, and the single-session design limits the generalizability of the findings. While diet and physical activity were controlled, residual confounding factors cannot be excluded. Crucially, no direct biomarkers of mitochondrial function (e.g., ATP production, cytochrome c oxidase activity, PGC-1α expression) were measured; therefore, any inference about mitochondrial efficiency remains hypothetical.

Future research should employ repeated PBM sessions within longitudinal randomized trials to evaluate chronic effects on energy metabolism, body composition, and cardiometabolic health. Studies integrating biochemical, molecular, and imaging measures of mitochondrial activity will be essential to elucidate the underlying mechanisms.

## 5. Conclusions

In summary, our findings demonstrate that a single 12-min session of PBM appeared to acutely increase the overall oxidative metabolic rate without modifying the balance between the substrates being oxidized. These findings suggest an acute modulation of energy metabolism rather than a confirmed improvement in mitochondrial efficiency. PBM appears safe and well-tolerated, supporting its potential as a non-invasive adjunct for metabolic health, pending confirmation from larger and mechanistically oriented studies.

## Figures and Tables

**Table 1:** Study-subjects’ characteristics.

	Normal-Weight	With Obesity	*p* Value
	(*n* = 16)	(*n =* 16)	
Age (years)	43.4 ± 4.8	43.4 ± 4.8	n.s.
Height (cm)	165 ± 4	163 ± 6	n.s.
Weight (kg)	62.6 ± 6.3	96.2 ± 18	<0.001
BMI (kg/m^2^)	22.7 ± 2	36 ± 4	<0.001
Total body fat (%)	27.7 ± 4	42.4 ± 4.2	<0.001
Total muscle mass (kg)	42.9 ± 3	52.3 ± 8.6	<0.001
Total body water (%)	53.4 ± 2.9	42.9 ± 3.1	<0.001
Bone mass (kg)	2.29 ± 0.15	2.84 ± 0.56	<0.001
Physical activity level	sedentary	sedentary	n.s.
Dietary intake (kcal/day)	1254 ± 191	1526 ± 1096	n.s.

Data are shown as mean ± SD.

**Table 2:** Study-subjects’ results throughout the different experimental session.

	Normal-Weight				With Obesity			
	SHAM		PBM		SHAM		PBM	
	Pre	Post	Pre	Post	Pre	Post	Pre	Post
VO_2_ (mL/min)	220 ± 50.6	227 ± 63.7	219 ± 38	231 ± 44.9	221 ± 39.3	219 ± 50.5	217 ± 47.3 ^a^	232 ± 48.7 ^a^
VCO_2_ (mL/min)	176 ± 46.4	172 ± 47	171 ± 33	171 ± 35	171 ± 40.2	163 ± 43	172 ± 42.7	177 ± 45
RER (VCO_2_/VO_2_)	0.78 ± 0.08	0.73 ± 0.06	0.77 ± 0.07	0.73 ± 0.06	0.77 ± 0.07	0.73 ± 0.06	0.79 ± 0.1	0.76 ± 0.08
REE (kcal/day)	1527 ± 324	1569 ± 391	1497 ± 264 ^a^	1573 ± 301 ^a^	1526 ± 273	1516 ± 312	1486 ± 327 ^b^	1624 ± 314 ^b^
HR (bpm)	81 ± 18 ^a^	70 ± 22 ^a^	76 ± 17	73 ± 14	89 ± 22 ^b^	80 ± 27 ^b^	87 ± 21	81 ± 22
Systolic pressure (mmHg)	105 ± 8	104 ± 9	102 ± 11	104 ± 12	132 ± 25	131 ± 22	129 ± 20	122 ± 17
POMS (a.u.)	95 ± 6	95 ± 7	97 ± 8 ^a^	93 ± 4 ^a^	98 ± 9 ^b^	94 ± 6 ^b^	99 ± 6 ^c^	94 ± 4 ^c^
RPE (a.u.)	8 ± 2	8 ± 2	10 ± 2 ^a^	8 ± 2 ^a^	11 ± 3	11 ± 2	11 ± 3 ^b^	8 ± 2 ^b^
Skin AF (a.u.)	1.8 ± 0.3	1.8 ± 0.3	1.8 ± 0.3	1.8 ± 0.3	2.4 ± 0.5	2.4 ± 0.5	2.2 ± 0.4	2.2 ± 0.4
Skin front temp. (°C)	32.3 ± 1	32.5 ± 1.1	32.4 ± 0.7 ^a^	35.6 ± 0.8 ^a^	31.6 ± 0.8	31.9 ± 0.7	31.6 ± 0.8 ^b^	34.4 ± 0.9 ^b^
Skin back temp. (°C)	32.3 ± 1	32.8 ± 1.1	32.4 ± 0.7 ^a^	37± 1.1 ^a^	31 ± 0.7	31 ± 0.6	31 ± 0.8 ^b^	36.1 ± 0.8 ^b^
Flexibility (cm)	8.37 ± 9	8.62 ± 8.78	7.5 ± 9.1 ^a^	9.93 ± 9.23 ^a^	0.5 ± 8.59	0.87 ± 8.49	0.06 ± 8.25 ^b^	2.18 ± 9.15 ^b^
Strength (kg)	26.6 ± 5.6	26.3 ± 6	23.9 ± 5.7	25.2 ± 5.9	27 ± 5.5	27 ± 5.8	25.7 ± 6.6	26.2 ± 6.8

Data are shown as mean ± SD. For time effect, significant pairwise comparisons within each row are indicated by the same letter (p < 0.05). Other significant effects are reported within the text.

## Data Availability

The original contributions presented in this study are included in the article. Further inquiries can be directed to the corresponding author.

## Supplementary Materials

The following supporting information can be downloaded at: https://www.mdpi.com/article/10.3390/nu17213357/s1, Figure S1: Frontal-lateral view of the red-light therapy bed with the lid slightly open. Figure S2: (A) Lateral view of the red-light therapy bed with the lid tightly closed; (B) Zoom-in of the lateral view showing the magnified distance between the light source (led stripes) and the contact surface (glass). Table S1: Pearson correlations between change in resting energy expenditure (ΔREE) and change in skin temperature (ΔT) at front and back exposure sites, by group and condition; Table S2: Estimated habitual dietary composition by group.

## Abbreviations

The following abbreviations are used in this manuscript:

ATP	Adenosine triphosphate
BMI	Body Mass Index
BP	Blood Pressure
HR	Heart Rate
PBM	Photobiomodulation
POMS	Profiles of Mood States
REE	Resting Energy Expenditure
RER	Respiratory Exchange Ratio
ROI	Region Of Interest
RPE	Rate of Perceived Exertion
