# Supplementary material for: Photobiomodulation Acutely Augments Resting Metabolism in Women with Obesity
Source: Nutrients. 2025 Oct 25;17(21):3357. doi: 10.3390/nu17213357 (PMC12608151; doi:10.3390/nu17213357)
Supplement: Supplementary file 1 [file nutrients-17-03357-s001.zip › Nutrients_Supplementary figures 1,2_rev.pdf]

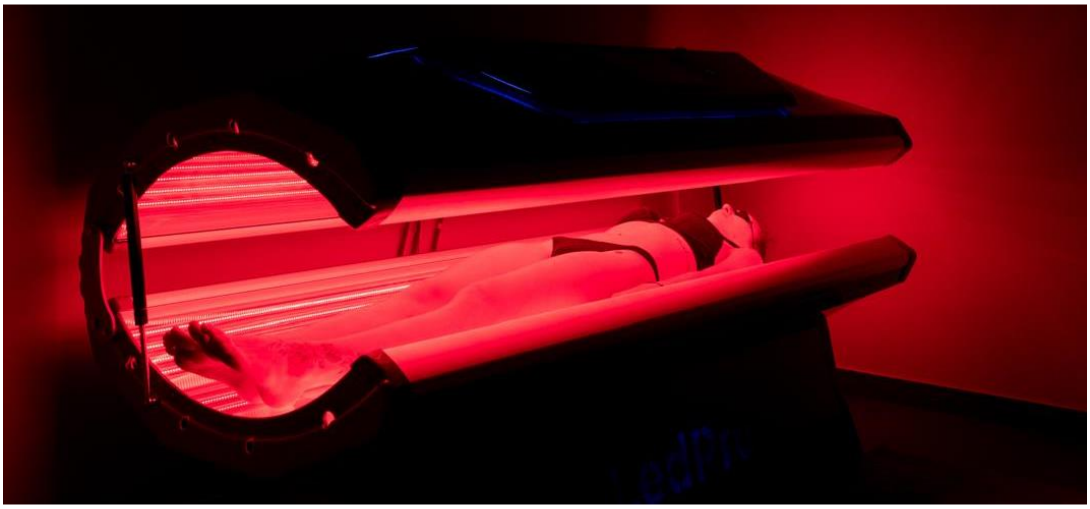

Figure S1. Frontal-lateral view of the red-light therapy bed with the lid slightly open.

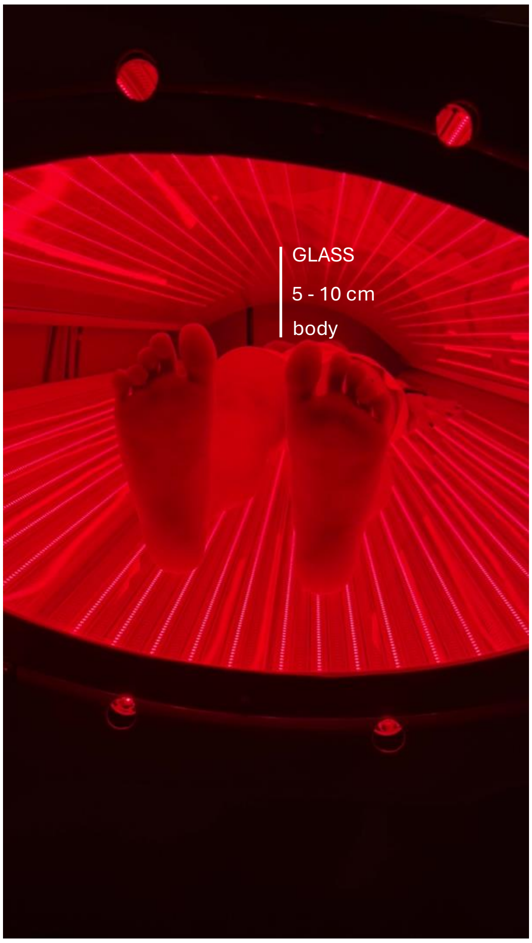

Figure S2 A. Lateral view of the red-light therapy bed with the lid tightly closed.

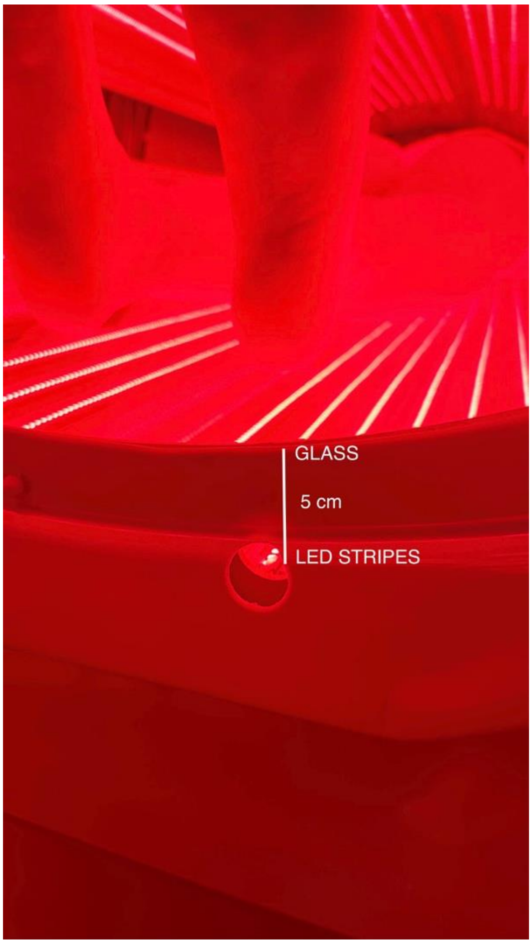

Figure S2 B. Zoom-in of the lateral view showing the magnified distance between the light source (led stripes) and the contact surface (glass).
