# Supplementary material for: Photobiomodulation Acutely Augments Resting Metabolism in Women with Obesity
Source: Nutrients. 2025 Oct 25;17(21):3357. doi: 10.3390/nu17213357 (PMC12608151; doi:10.3390/nu17213357)
Supplement: Supplementary file 1 [file nutrients-17-03357-s001.zip › Supplementary Table S1.pdf]

Supplementary Table S1. Pearson correlations between change in resting energy expenditure (ΔREE) and change in skin temperature (ΔT) at front and back exposure sites, by group and condition.

Group	Condition	Site	n	r	95% CI (lower–upper)	p-value
Normal-weight	SHAM	Front	16	0.4950	0.1408 – 0.5423	0.0681
Normal-weight	SHAM	Back	16	0.4830	-0.01668 – 0.7897	0.0581
Obesity	SHAM	Front	16	-0.3030	-0.6944 – 0.2268	0.2540
Obesity	SHAM	Back	16	-0.3219	-0.7051 – 0.2068	0.2240
Normal-weight	PBM	Front	16	0.2356	-0.2945 – 0.6548	0.3797
Normal-weight	PBM	Back	16	0.3627	-0.1621 – 0.7276	0.1673
Obesity	PBM	Front	16	-0.1035	-0.5700 – 0.4134	0.7029
Obesity	PBM	Back	16	0.2046	-0.3239 – 0.6358	0.4472
