# Supplementary material for: Photobiomodulation Acutely Augments Resting Metabolism in Women with Obesity
Source: Nutrients. 2025 Oct 25;17(21):3357. doi: 10.3390/nu17213357 (PMC12608151; doi:10.3390/nu17213357)
Supplement: Supplementary file 1 [file nutrients-17-03357-s001.zip › Supplementary_Table_S2_Dietary_Composition.pdf]

Supplementary Table S2. Estimated habitual dietary composition by group

Habitual dietary composition estimated from EPIC-based Food Frequency Questionnaire (FETA) outputs for women with obesity (n = 16) and normal-weight women (n = 16). Values are expressed as mean ± SD. TEI = total energy intake.

Parameter	Women with obesity (n=16)	Normal-weight women (n=16)
Total Energy Intake (kcal/day)	1,526 ± 1096	1,254 ± 191
Protein (g/day, %TEI)	66 ± 14 (20%)	76 ± 16 (17%)
Total Fat (g/day, %TEI)	57 ± 42 (39%)	70 ± 14 (35%)
Carbohydrates (g/day, %TEI)	155 ± 82 (41%)	210 ± 40 (46%)
Fiber (g/day)	13 ± 5	21 ± 6
Alcohol (g/day)	2 ± 4	4 ± 5
Cholesterol (mg/day)	300 ± 90	280 ± 70
Sodium (g/day)	2.5 ± 0.7	2.3 ± 0.6
Main food contributors	Bread > meat > dairy > sweets > vegetables	Bread > vegetables > fruit > dairy > meat

*Values estimated from the FETA software (EPIC-based dietary questionnaire) and representative European reference datasets for adult women aged 40–50 years. Dietary intake was consistent across conditions (PBM vs SHAM).
